# *catena*-Poly[[aqua­(dipyrido[3,2-*a*:2′,3′-*c*]phenazine-κ^2^ *N* ^4^,*N* ^5^)zinc(II)]-μ-pyrazine-2,3-dicarboxyl­ato-κ^3^ *N* ^1^,*O* ^2^:*O* ^3^]

**DOI:** 10.1107/S1600536808022824

**Published:** 2008-07-26

**Authors:** Xin Wang, Xiu-Ying Li, Qing-Wei Wang, Guang-Bo Che

**Affiliations:** a Jilin Agriculture Engineering Polytechnic College, Siping 136000, People’s Republic of China; b Department of Chemistry, Jilin Normal University, Siping 136000, People’s Republic of China

## Abstract

In the title compound, [Zn(C_6_H_2_N_2_O_4_)(C_18_H_10_N_4_)(H_2_O)]_*n*_ or [Zn(PZDC)(DPPZ)(H_2_O)]_*n*_ (where DPPZ is dipyrido[3,2-*a*:2′,3′-*c*]phenazine and H_2_PZDC is pyrazine-2,3-dicarboxylic acid), the Zn atom is six-coordinated in a slightly distorted octa­hedral coordination geometry by three N atoms from one DPPZ ligand and one PZDC^2−^ dianion, three O atoms from two different PZDC^2−^ ligands and one water mol­ecule. Each PZDC^2−^ dianion serves as a spacer, connecting adjacent metal atoms into a polymeric chain structure parallel to the *b* axis. The chain motif is consolidated into a three-dimensional supra­molecular network by O—H⋯O and O—H⋯N hydrogen bonds and π–π aromatic stacking inter­actions involving adjacent DPPZ ligands and PZDC^2−^ dianions with centroid–centroid separations of 3.522 (6) and 3.732 (8) Å, respectively.

## Related literature

For related literature, see: Che *et al.* (2008 ▶); Che, Li *et al.* (2006 ▶); Che, Xu & Liu (2006 ▶); Liu *et al.* (2008 ▶); Xu *et al.* (2008 ▶).
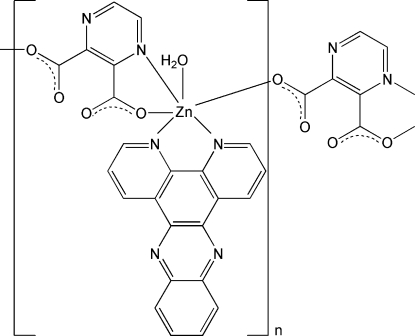


## Experimental

### 

#### Crystal data

[Zn(C_6_H_2_N_2_O_4_)(C_18_H_10_N_4_)(H_2_O)]
*M* *_r_* = 531.78
Triclinic, 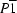

*a* = 6.7821 (14) Å
*b* = 7.4349 (15) Å
*c* = 20.410 (4) Å
α = 91.26 (3)°
β = 95.77 (3)°
γ = 98.16 (3)°
*V* = 1012.9 (4) Å^3^
*Z* = 2
Mo *K*α radiation
μ = 1.27 mm^−1^
*T* = 292 (2) K
0.31 × 0.29 × 0.21 mm

#### Data collection

Rigaku R-AXIS RAPID diffractometer
Absorption correction: multi-scan (*ABSCOR*; Higashi, 1995 ▶) *T*_min_ = 0.681, *T*_max_ = 0.765
9890 measured reflections
4412 independent reflections
3278 reflections with *I* > 2σ(*I*)
*R*_int_ = 0.048

#### Refinement

*R*[*F* ^2^ > 2σ(*F* ^2^)] = 0.048
*wR*(*F* ^2^) = 0.148
*S* = 1.07
4412 reflections
333 parameters
H atoms treated by a mixture of independent and constrained refinement
Δρ_max_ = 0.42 e Å^−3^
Δρ_min_ = −0.58 e Å^−3^

### 

Data collection: *PROCESS-AUTO* (Rigaku, 1998 ▶); cell refinement: *PROCESS-AUTO*; data reduction: *PROCESS-AUTO*; program(s) used to solve structure: *SHELXS97* (Sheldrick, 2008 ▶); program(s) used to refine structure: *SHELXL97* (Sheldrick, 2008 ▶); molecular graphics: *SHELXTL-Plus* (Sheldrick, 2008 ▶); software used to prepare material for publication: *SHELXL97*.

## Supplementary Material

Crystal structure: contains datablocks global, I. DOI: 10.1107/S1600536808022824/rz2235sup1.cif

Structure factors: contains datablocks I. DOI: 10.1107/S1600536808022824/rz2235Isup2.hkl

Additional supplementary materials:  crystallographic information; 3D view; checkCIF report

## Figures and Tables

**Table:** 

N1—Zn	2.130 (3)
N2—Zn	2.167 (3)
N5—Zn	2.147 (3)
O1—Zn	2.172 (3)
O1*W*—Zn	2.120 (3)
O4—Zn^i^	2.051 (3)

**Table:** 

O4^ii^—Zn—O1*W*	90.19 (13)
O4^ii^—Zn—N1	90.37 (12)
O1*W*—Zn—N1	96.93 (13)
O4^ii^—Zn—N5	97.78 (12)
O1*W*—Zn—N5	86.87 (13)
N1—Zn—N5	171.02 (11)
O4^ii^—Zn—N2	98.61 (11)
O1*W*—Zn—N2	169.43 (13)

Symmetry codes: (i) 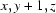; (ii) 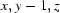.

**Table 2:** Hydrogen-bond geometry (Å, °)

*D*—H⋯*A*	*D*—H	H⋯*A*	*D*⋯*A*	*D*—H⋯*A*
O1*W*—H*W*1*A*⋯O3^iii^	0.66 (5)	2.01 (5)	2.662 (4)	169 (7)
O1*W*—H*W*1*B*⋯N6^iv^	0.82 (5)	2.07 (5)	2.859 (5)	159 (4)

Symmetry codes: (iii) 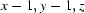; (iv) 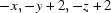.

**Fig. 1.**
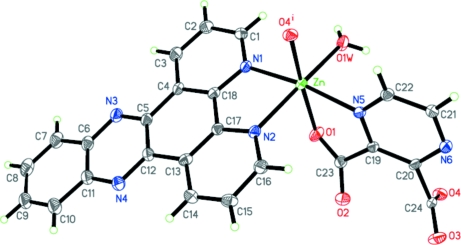
A view of the title compound. Displacement ellipsoids are drawn at the 30% probability level (arbitrary spheres for the H atoms). [Symmetry code: (i) x, y + 1, z.]

**Fig. 2.**
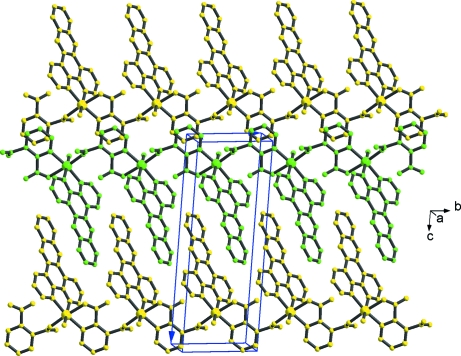
Packing diagram of the three-dimensional supramolecular structure of the title compound formed viaπ-π interactions. H atoms are omitted for clarity.

## supplementary crystallographic information

### Comment

A successful strategy for preparing metal-organic supramolecular architectures is the assembly reaction between a transition d^10^ metal ion and two types of ligands with one acting as a bridging ligand and the other as a chelating ligand (Liu *et al.*, 2008; Che *et al.*, 2008). Pyrazine-2,3-dicarboxylic acid (H_2_PZDC) possesses the ability to bridge and chelate metal atoms using the carboxylate oxygen atoms and nitrogen atoms (Xu *et al.*, 2008). 1,10-Phenanthroline (phen) and its derivatives are important chelating ligands for the construction of metal-organic complexes (Che, Xu & Liu, 2006). Dipyrido[3,2-a:2',3'-c]-phenazine (DPPZ) as a phen derivative possesses potential supramolecular recognition sites for π-π aromatic stacking interactions. The present attempt at synthesizing a new zinc polymer with DPPZ and H_2_PZDC gave the title complex, [Zn(DPPZ)(PZDC)(H_2_O)]_n_, whose structure is reported here.

The Zn atom is six-coordinated by three N atoms from one DPPZ ligand and one PZDC^2-^ ligand, and three O atoms from two different PZDC^2-^ ligands and one water molecule in a slightly distorted octahedral coordination geometry (Fig. 1). The Zn—O distances range from 2.051 (3) Å to 2.172 (3) Å and the Zn—N lengths from 2.130 (3) Å to 2.167 (3) Å (Table 1). Each PZDC^2-^ dianion serves as a spacer to connect adjacent metal centres into a one-dimensional chain structure parallel to the *b* axis. Neighbouring chains interact through π-π contacts, leading to a three-dimensional supramolecular structure (Fig. 2). There are two types of π-π interactions, occurring between adjacent DPPZ ligands (centroid-to-centroid separation = 3.732 (8) Å) and PZDC^2-^ anions (centroid-to-centroid separation = 3.522 (6) Å). Hydrogen bonds involving the O1W atom as donor and the N6 and O3 atoms of the PZDC^2-^ dianion as acceptors further stabilize the structure (Table 2).

### Experimental

The DPPZ ligand was synthesized according to the literature method (Che, Li *et al.*, 2006). The title compound was hydrothermally synthesized under autogenous pressure: a mixture of DPPZ, H_2_PZDC, ZnNO_3_ and water in a molar ratio of 1:1:1:5000 was sealed in a Teflon-lined autoclave and heated to 433 K for 3 d. Upon cooling and opening the bomb, yellow blocks of the title compound were obtained (83% yield based on Zn).

### Refinement

All H atoms on C atoms were positioned geometrically (C—H = 0.93 Å) and refined as riding, with *U*_iso_(H)= 1.2*U*_eq_(C). The hydrogen atoms of water molecule were located from a difference Fourier map and refined freely.

### Crystal data

**Table:**

[Zn(C_6_H_2_N_2_O_4_)(C_18_H_10_N_4_)(H_2_O)]	*Z* = 2
*M**_r_* = 531.78	*F*_000_ = 540
Triclinic, *P*1	*D*_x_ = 1.744 Mg m^−^^3^
Hall symbol: -P 1	Mo *K*α radiation λ = 0.71073 Å
*a* = 6.7821 (14) Å	Cell parameters from 4412 reflections
*b* = 7.4349 (15) Å	θ = 3.0–27.5º
*c* = 20.410 (4) Å	µ = 1.27 mm^−^^1^
α = 91.26 (3)º	*T* = 292 (2) K
β = 95.77 (3)º	Block, yellow
γ = 98.16 (3)º	0.31 × 0.29 × 0.21 mm
*V* = 1012.9 (4) Å^3^	

### Data collection

**Table:**

Rigaku R-AXIS RAPID diffractometer	4412 independent reflections
Radiation source: fine-focus sealed tube	3278 reflections with *I* > 2σ(*I*)
Monochromator: graphite	*R*_int_ = 0.048
Detector resolution: 10.0 pixels mm^-1^	θ_max_ = 27.5º
*T* = 292(2) K	θ_min_ = 3.0º
ω scans	*h* = −8→8
Absorption correction: multi-scan(ABSCOR; Higashi, 1995)	*k* = −9→8
*T*_min_ = 0.681, *T*_max_ = 0.765	*l* = −26→26
9890 measured reflections	

### Refinement

**Table:**

Refinement on *F*^2^	Secondary atom site location: difference Fourier map
Least-squares matrix: full	Hydrogen site location: inferred from neighbouring sites
*R*[*F*^2^ > 2σ(*F*^2^)] = 0.048	H atoms treated by a mixture of independent and constrained refinement
*wR*(*F*^2^) = 0.148	*w* = 1/[σ^2^(*F*_o_^2^) + (0.084*P*)^2^ + 0.1727*P*] where *P* = (*F*_o_^2^ + 2*F*_c_^2^)/3
*S* = 1.07	(Δ/σ)_max_ < 0.001
4412 reflections	Δρ_max_ = 0.42 e Å^−^^3^
333 parameters	Δρ_min_ = −0.58 e Å^−^^3^
Primary atom site location: structure-invariant direct methods	Extinction correction: none

### Special details

**Table:**

Geometry. All e.s.d.'s (except the e.s.d. in the dihedral angle between two l.s. planes) are estimated using the full covariance matrix. The cell e.s.d.'s are taken into account individually in the estimation of e.s.d.'s in distances, angles and torsion angles; correlations between e.s.d.'s in cell parameters are only used when they are defined by crystal symmetry. An approximate (isotropic) treatment of cell e.s.d.'s is used for estimating e.s.d.'s involving l.s. planes.
Refinement. Refinement of *F*^2^ against ALL reflections. The weighted *R*-factor *wR* and goodness of fit *S* are based on *F*^2^, conventional *R*-factors *R* are based on *F*, with *F* set to zero for negative *F*^2^. The threshold expression of *F*^2^ > σ(*F*^2^) is used only for calculating *R*-factors(gt) *etc*. and is not relevant to the choice of reflections for refinement. *R*-factors based on *F*^2^ are statistically about twice as large as those based on *F*, and *R*- factors based on ALL data will be even larger.

### Fractional atomic coordinates and isotropic or equivalent isotropic displacement parameters (Å^2^)

**Table:**

	*x*	*y*	*z*	*U*_iso_*/*U*_eq_	
C1	−0.3785 (6)	0.2965 (5)	0.7826 (2)	0.0344 (9)	
H1	−0.4426	0.3029	0.8206	0.041*	
C2	−0.4723 (6)	0.1853 (6)	0.7307 (2)	0.0375 (10)	
H2	−0.5931	0.1119	0.7349	0.045*	
C3	−0.3868 (6)	0.1832 (6)	0.6728 (2)	0.0395 (10)	
H3	−0.4528	0.1144	0.6364	0.047*	
C4	−0.1986 (6)	0.2861 (5)	0.6690 (2)	0.0291 (8)	
C5	−0.0963 (6)	0.2913 (5)	0.60945 (19)	0.0283 (8)	
C6	−0.0924 (7)	0.2081 (5)	0.5015 (2)	0.0352 (9)	
C7	−0.1870 (8)	0.1180 (6)	0.4421 (2)	0.0443 (11)	
H7	−0.3185	0.0601	0.4400	0.053*	
C8	−0.0836 (8)	0.1170 (6)	0.3880 (2)	0.0493 (12)	
H8	−0.1458	0.0582	0.3491	0.059*	
C9	0.1164 (9)	0.2037 (6)	0.3903 (2)	0.0514 (12)	
H9	0.1845	0.2003	0.3531	0.062*	
C10	0.2107 (8)	0.2921 (7)	0.4464 (2)	0.0479 (12)	
H10	0.3420	0.3496	0.4471	0.058*	
C11	0.1095 (7)	0.2967 (6)	0.5036 (2)	0.0379 (10)	
C12	0.1042 (6)	0.3850 (5)	0.61078 (19)	0.0292 (8)	
C13	0.1983 (6)	0.4856 (5)	0.67084 (19)	0.0286 (8)	
C14	0.3909 (6)	0.5847 (6)	0.6747 (2)	0.0345 (9)	
H14	0.4637	0.5883	0.6384	0.041*	
C15	0.4710 (6)	0.6763 (6)	0.7322 (2)	0.0346 (9)	
H15	0.5979	0.7444	0.7353	0.041*	
C16	0.3603 (6)	0.6662 (5)	0.7859 (2)	0.0326 (9)	
H16	0.4165	0.7264	0.8253	0.039*	
C17	0.0958 (5)	0.4841 (5)	0.72651 (18)	0.0242 (7)	
C18	−0.1092 (5)	0.3868 (5)	0.72530 (18)	0.0256 (8)	
C19	0.1302 (5)	0.9551 (5)	0.89690 (18)	0.0237 (7)	
C20	0.1992 (5)	1.1069 (5)	0.93824 (19)	0.0247 (8)	
C21	0.2685 (6)	0.9238 (5)	1.02315 (19)	0.0288 (8)	
H21	0.3121	0.9090	1.0671	0.035*	
C22	0.2102 (5)	0.7712 (5)	0.98204 (19)	0.0261 (8)	
H22	0.2235	0.6566	0.9978	0.031*	
C23	0.0378 (6)	0.9589 (5)	0.82528 (19)	0.0271 (8)	
C24	0.2040 (5)	1.3019 (5)	0.91602 (18)	0.0249 (8)	
N1	−0.1984 (5)	0.3963 (4)	0.78077 (16)	0.0267 (7)	
N2	0.1761 (5)	0.5730 (4)	0.78317 (15)	0.0271 (7)	
N3	−0.1925 (5)	0.2047 (4)	0.55512 (17)	0.0349 (8)	
N4	0.2041 (5)	0.3872 (5)	0.55828 (17)	0.0348 (8)	
N5	0.1356 (4)	0.7877 (4)	0.92032 (15)	0.0234 (6)	
N6	0.2640 (5)	1.0909 (4)	1.00185 (16)	0.0281 (7)	
O1	−0.0880 (4)	0.8227 (4)	0.80655 (14)	0.0362 (7)	
O2	0.0974 (5)	1.0921 (4)	0.79424 (15)	0.0410 (7)	
O1W	−0.2753 (5)	0.5957 (5)	0.91335 (17)	0.0389 (8)	
O3	0.3705 (4)	1.3872 (4)	0.91050 (17)	0.0427 (8)	
O4	0.0381 (4)	1.3587 (3)	0.90882 (14)	0.0305 (6)	
Zn	−0.03443 (6)	0.57612 (5)	0.85620 (2)	0.02642 (16)	
HW1A	−0.368 (8)	0.552 (8)	0.909 (3)	0.054 (19)*	
HW1B	−0.295 (7)	0.692 (7)	0.930 (2)	0.034 (12)*	

### Atomic displacement parameters (Å^2^)

**Table:**

	*U*^11^	*U*^22^	*U*^33^	*U*^12^	*U*^13^	*U*^23^
C1	0.034 (2)	0.031 (2)	0.037 (2)	−0.0052 (17)	0.0120 (17)	−0.0021 (17)
C2	0.032 (2)	0.036 (2)	0.042 (2)	−0.0062 (18)	0.0058 (18)	−0.0039 (18)
C3	0.038 (2)	0.037 (2)	0.038 (2)	−0.0082 (19)	0.0014 (18)	−0.0108 (18)
C4	0.0326 (19)	0.0232 (18)	0.030 (2)	−0.0004 (15)	0.0040 (16)	−0.0055 (15)
C5	0.0333 (19)	0.0250 (18)	0.0254 (19)	0.0033 (16)	−0.0004 (15)	−0.0022 (15)
C6	0.047 (2)	0.032 (2)	0.027 (2)	0.0109 (19)	−0.0004 (18)	−0.0010 (16)
C7	0.058 (3)	0.040 (2)	0.032 (2)	0.008 (2)	−0.006 (2)	−0.0054 (18)
C8	0.080 (4)	0.040 (2)	0.026 (2)	0.015 (2)	−0.011 (2)	−0.0046 (18)
C9	0.082 (4)	0.043 (3)	0.032 (2)	0.011 (3)	0.019 (2)	−0.003 (2)
C10	0.065 (3)	0.048 (3)	0.033 (2)	0.011 (2)	0.013 (2)	−0.001 (2)
C11	0.050 (2)	0.034 (2)	0.030 (2)	0.007 (2)	0.0097 (19)	−0.0021 (17)
C12	0.0334 (19)	0.0291 (19)	0.025 (2)	0.0046 (16)	0.0045 (16)	−0.0019 (15)
C13	0.0273 (18)	0.0256 (19)	0.031 (2)	−0.0009 (15)	−0.0011 (15)	0.0024 (15)
C14	0.033 (2)	0.037 (2)	0.035 (2)	0.0046 (17)	0.0107 (17)	0.0014 (17)
C15	0.0257 (18)	0.039 (2)	0.037 (2)	−0.0023 (17)	0.0029 (17)	0.0059 (18)
C16	0.032 (2)	0.034 (2)	0.029 (2)	−0.0023 (17)	−0.0001 (16)	−0.0016 (16)
C17	0.0242 (17)	0.0212 (17)	0.0275 (19)	0.0049 (14)	0.0029 (14)	0.0012 (14)
C18	0.0274 (18)	0.0208 (17)	0.0273 (19)	0.0009 (14)	−0.0003 (15)	0.0009 (14)
C19	0.0223 (16)	0.0213 (17)	0.0269 (19)	−0.0007 (14)	0.0061 (14)	−0.0005 (14)
C20	0.0207 (16)	0.0185 (17)	0.034 (2)	−0.0023 (14)	0.0084 (15)	−0.0024 (15)
C21	0.0315 (18)	0.0289 (19)	0.0245 (19)	−0.0020 (16)	0.0060 (15)	−0.0003 (15)
C22	0.0281 (18)	0.0223 (17)	0.029 (2)	0.0052 (15)	0.0045 (15)	0.0031 (15)
C23	0.0302 (18)	0.0231 (18)	0.028 (2)	0.0050 (15)	0.0032 (15)	0.0015 (15)
C24	0.0307 (18)	0.0192 (16)	0.0228 (18)	−0.0046 (15)	0.0066 (15)	−0.0052 (13)
N1	0.0270 (15)	0.0228 (15)	0.0294 (17)	−0.0004 (13)	0.0050 (13)	−0.0041 (12)
N2	0.0301 (16)	0.0242 (15)	0.0240 (16)	−0.0034 (13)	−0.0008 (13)	−0.0030 (12)
N3	0.0423 (19)	0.0304 (17)	0.0305 (19)	0.0023 (15)	0.0017 (15)	−0.0043 (14)
N4	0.0417 (19)	0.0354 (18)	0.0265 (18)	0.0027 (15)	0.0038 (15)	−0.0016 (14)
N5	0.0243 (14)	0.0201 (14)	0.0260 (16)	0.0017 (12)	0.0060 (12)	0.0013 (12)
N6	0.0277 (15)	0.0260 (16)	0.0294 (17)	−0.0008 (13)	0.0049 (13)	−0.0033 (13)
O1	0.0410 (15)	0.0258 (14)	0.0365 (16)	−0.0014 (12)	−0.0119 (13)	0.0002 (12)
O2	0.0549 (19)	0.0329 (15)	0.0347 (17)	0.0005 (14)	0.0095 (14)	0.0072 (12)
O1W	0.0271 (16)	0.0357 (18)	0.052 (2)	−0.0066 (14)	0.0164 (14)	−0.0192 (15)
O3	0.0335 (15)	0.0341 (16)	0.057 (2)	−0.0112 (13)	0.0087 (14)	0.0051 (14)
O4	0.0342 (14)	0.0237 (13)	0.0367 (16)	0.0096 (11)	0.0091 (12)	0.0088 (11)
Zn	0.0313 (3)	0.0203 (2)	0.0259 (3)	−0.00162 (17)	0.00328 (17)	−0.00223 (16)

### Geometric parameters (Å, °)

**Table:**

C1—N1	1.341 (5)	C15—H15	0.9300
C1—C2	1.374 (6)	C16—N2	1.335 (5)
C1—H1	0.9300	C16—H16	0.9300
C2—C3	1.369 (6)	C17—N2	1.345 (5)
C2—H2	0.9300	C17—C18	1.471 (5)
C3—C4	1.402 (5)	C18—N1	1.341 (5)
C3—H3	0.9300	C19—N5	1.347 (4)
C4—C18	1.393 (5)	C19—C20	1.390 (5)
C4—C5	1.457 (5)	C19—C23	1.534 (5)
C5—N3	1.332 (5)	C20—N6	1.342 (5)
C5—C12	1.435 (5)	C20—C24	1.525 (5)
C6—N3	1.344 (5)	C21—N6	1.328 (5)
C6—C7	1.420 (6)	C21—C22	1.383 (5)
C6—C11	1.429 (6)	C21—H21	0.9300
C7—C8	1.367 (7)	C22—N5	1.325 (5)
C7—H7	0.9300	C22—H22	0.9300
C8—C9	1.412 (7)	C23—O2	1.229 (4)
C8—H8	0.9300	C23—O1	1.253 (5)
C9—C10	1.360 (7)	C24—O3	1.231 (4)
C9—H9	0.9300	C24—O4	1.254 (5)
C10—C11	1.414 (6)	N1—Zn	2.130 (3)
C10—H10	0.9300	N2—Zn	2.167 (3)
C11—N4	1.346 (5)	N5—Zn	2.147 (3)
C12—N4	1.324 (5)	O1—Zn	2.172 (3)
C12—C13	1.462 (5)	O1W—Zn	2.120 (3)
C13—C17	1.390 (5)	O1W—HW1A	0.66 (5)
C13—C14	1.399 (5)	O1W—HW1B	0.82 (5)
C14—C15	1.367 (6)	O4—Zn^i^	2.051 (3)
C14—H14	0.9300	Zn—O4^ii^	2.051 (3)
C15—C16	1.387 (6)		
			
N1—C1—C2	122.7 (4)	N1—C18—C17	116.8 (3)
N1—C1—H1	118.6	C4—C18—C17	119.9 (3)
C2—C1—H1	118.6	N5—C19—C20	119.5 (3)
C3—C2—C1	119.5 (4)	N5—C19—C23	115.0 (3)
C3—C2—H2	120.2	C20—C19—C23	125.5 (3)
C1—C2—H2	120.2	N6—C20—C19	121.5 (3)
C2—C3—C4	119.2 (4)	N6—C20—C24	115.0 (3)
C2—C3—H3	120.4	C19—C20—C24	123.5 (3)
C4—C3—H3	120.4	N6—C21—C22	122.1 (4)
C18—C4—C3	117.3 (4)	N6—C21—H21	119.0
C18—C4—C5	119.9 (3)	C22—C21—H21	119.0
C3—C4—C5	122.9 (4)	N5—C22—C21	120.2 (3)
N3—C5—C12	121.8 (4)	N5—C22—H22	119.9
N3—C5—C4	118.3 (3)	C21—C22—H22	119.9
C12—C5—C4	120.0 (3)	O2—C23—O1	129.1 (4)
N3—C6—C7	119.4 (4)	O2—C23—C19	116.4 (3)
N3—C6—C11	121.2 (4)	O1—C23—C19	114.5 (3)
C7—C6—C11	119.3 (4)	O3—C24—O4	127.8 (3)
C8—C7—C6	119.6 (5)	O3—C24—C20	116.4 (3)
C8—C7—H7	120.2	O4—C24—C20	115.7 (3)
C6—C7—H7	120.2	C1—N1—C18	117.8 (3)
C7—C8—C9	121.0 (4)	C1—N1—Zn	127.4 (3)
C7—C8—H8	119.5	C18—N1—Zn	114.9 (2)
C9—C8—H8	119.5	C16—N2—C17	118.8 (3)
C10—C9—C8	120.8 (5)	C16—N2—Zn	127.2 (3)
C10—C9—H9	119.6	C17—N2—Zn	113.4 (2)
C8—C9—H9	119.6	C5—N3—C6	116.8 (4)
C9—C10—C11	120.1 (5)	C12—N4—C11	117.0 (4)
C9—C10—H10	119.9	C22—N5—C19	119.2 (3)
C11—C10—H10	119.9	C22—N5—Zn	126.6 (2)
N4—C11—C10	119.3 (4)	C19—N5—Zn	113.1 (2)
N4—C11—C6	121.5 (4)	C21—N6—C20	117.3 (3)
C10—C11—C6	119.2 (4)	C23—O1—Zn	113.8 (2)
N4—C12—C5	121.6 (4)	Zn—O1W—HW1A	129 (5)
N4—C12—C13	119.0 (3)	Zn—O1W—HW1B	123 (3)
C5—C12—C13	119.3 (3)	HW1A—O1W—HW1B	100 (6)
C17—C13—C14	118.0 (4)	C24—O4—Zn^i^	127.6 (2)
C17—C13—C12	119.8 (3)	O4^ii^—Zn—O1W	90.19 (13)
C14—C13—C12	122.2 (4)	O4^ii^—Zn—N1	90.37 (12)
C15—C14—C13	119.5 (4)	O1W—Zn—N1	96.93 (13)
C15—C14—H14	120.2	O4^ii^—Zn—N5	97.78 (12)
C13—C14—H14	120.2	O1W—Zn—N5	86.87 (13)
C14—C15—C16	119.0 (4)	N1—Zn—N5	171.02 (11)
C14—C15—H15	120.5	O4^ii^—Zn—N2	98.61 (11)
C16—C15—H15	120.5	O1W—Zn—N2	169.43 (13)
N2—C16—C15	122.4 (4)	N1—Zn—N2	77.29 (11)
N2—C16—H16	118.8	N5—Zn—N2	97.65 (12)
C15—C16—H16	118.8	O4^ii^—Zn—O1	174.40 (11)
N2—C17—C13	122.3 (3)	O1W—Zn—O1	90.70 (13)
N2—C17—C18	116.9 (3)	N1—Zn—O1	95.01 (11)
C13—C17—C18	120.8 (3)	N5—Zn—O1	76.76 (11)
N1—C18—C4	123.3 (3)	N2—Zn—O1	81.10 (12)

Symmetry codes: (i) *x*, *y*+1, *z*; (ii) *x*, *y*−1, *z*.

### Hydrogen-bond geometry (Å, °)

**Table:**

*D*—H···*A*	*D*—H	H···*A*	*D*···*A*	*D*—H···*A*
O1W—HW1A···O3^iii^	0.66 (5)	2.01 (5)	2.662 (4)	169 (7)
O1W—HW1B···N6^iv^	0.82 (5)	2.07 (5)	2.859 (5)	159 (4)

Symmetry codes: (iii) *x*−1, *y*−1, *z*; (iv) −*x*, −*y*+2, −*z*+2.
